# Two patients with a neuroendocrine tumour of the small intestine and paraneoplastic myasthenia gravis

**DOI:** 10.1530/EDM-14-0013

**Published:** 2014-05-01

**Authors:** M A W Hermans, B M L Stelten, H R Haak, W W de Herder, M W Dercksen

**Affiliations:** Department of Internal MedicineMaxima Medical CentreDs Theodor Fliednerstr 1; 5631BM; EindhovenThe Netherlands; 1 Department of NeurologyCanisius Wilhelmina HospitalWeg door Jonkerbos 100, 6532SZ, NijmegenThe Netherlands; 2 Department of Internal MedicineErasmus University Medical CentreGravendijkwal 230, 3015CE, RotterdamThe Netherlands

## Abstract

This paper reports on two patients with a long-standing diagnosis of an ENETS stage IV neuroendocrine tumour (NET) of the small intestine who developed neurological symptoms. The first patient only had bulbar symptoms and tested positive for acetylcholine receptor antibodies. The second patient had more classical symptoms of fatigable diplopia and muscle weakness of the legs, but no detectable antibodies. The diagnosis of paraneoplastical myasthenia gravis (MG) was postulated. Both patients were treated with pyridostigmine for MG and octreotide for the NETs. Interestingly, treatment of the NETs resulted in improvement of myasthenic symptoms. Paraneoplastic MG has been described to occur with certain malignancies, mainly thymoma. Herein, we prove that the association with gastrointestinal NETs, however, rare, is also one to be considered by clinicians dealing with either of these diseases. The pathogenesis has yet to be elucidated.

**Learning points:**

- NETs are rare malignancies with a wide variety of symptoms.
- Paraneoplastic MG can occur with various types of malignancies.
- Herein, we provide evidence of paraneoplastic MG in association with a grade IV NET of the small intestine.
- Treatment of the NETs resulted in remission of myasthenic symptoms in one patient.

## Background

Neuroendocrine tumours (NETs) are rare malignancies with a wide variety of symptoms. In this paper, we report on two patients with an ENETS stage IV NET of the small intestine, who develop paraneoplastic myasthenia gravis (MG) in concurrence with progression of their NETs. This is a rare autoimmune phenomenon related to gastrointestinal NETs. To our knowledge, this association was described only once before in the current literature. There are few other case reports of associated MG with NETs of the lung and pancreas (1) (2). Therefore, it is important for clinicians, dealing with either of these diseases, to be aware that MG can occur as a paraneoplastic phenomenon with NETs.

## Case presentation

### Patient A

The first patient is a 72-year-old Caucasian male who was diagnosed with a WHO grade I NET of the ileum for which partial resection of the ileum was performed in 1995. At that time, hepatic and mesenterial metastases were already present. Owing to the indolent behaviour, including stable 5-HIAA production, he was only treated symptomatically with colestyramine for diarrhoea. He did not suffer from other features of the carcinoid syndrome. The patient did not have any other relevant medical conditions. The follow-up was unremarkable with very slowly growing intra-abdominal metastases until March 2012, when the patient presented with progressive complaints of dysarthria and dysphagia that had developed over a period of 3 weeks. He denied having any visual difficulties. The dysarthria worsened when involved in a long conversation. Physical examination only revealed dysarthria and an ineffective swallowing reflex. Magnetic resonance imaging of the brain did not show any abnormalities. The diagnosis of (bulbar) MG was postulated on the basis of positive (>2.0 nmol/l) anti-acetylcholine receptor (AChR) antibodies and a declining response following repetitive nerve stimulation during electromyography (EMG). Accordingly, he was treated with pyridostigmine and a partial clinical response was achieved. CT scan of the chest did not show a thymic mass.

### Patient B

Our second patient is a 61-year-old Caucasian male with a WHO grade II, ENETS stage IV NET of unknown primary tumour site (most likely ileum) with mesenterial and lymph node metastases since 2008. He was steadily treated with octreotide analogues since 2009 and only had occasional diarrhoea and flushing. His medical history was otherwise not relevant. In January 2012, he first presented with walking difficulties, worsening by exercise. Neurological examination did not show any abnormalities at that time. Chromogranine A and urine 5-HIAA levels were 294 μg/l and 62.4 μmol/24 h respectively. In August 2012, hepatic metastases were diagnosed, and 3 months later, the patient presented with progressive symptoms of diplopia. Neurological examination elicited diplopia, but no other abnormalities including ptosis. Based on these fatigable symptoms, MG was suspected. However, assays for AChR antibodies, muscle-specific receptor tyrosine kinase (MuSK) antibodies and voltage-gated calcium channel (VGCC) antibodies were all negative. Single-fibre EMG was also unremarkable. Although the diagnosis of MG could not be confirmed with EMG, the fatigable aspect of his symptoms was very suggestive for myasthenia, and therefore, the patient was started on pyridostigmine 60 mg three times daily. This led to a remarkable decrease in symptoms, which leads us to believe that the patient also did suffer from MG.

## Outcome and follow-up

In patient A, simultaneously with the diagnosis of MG, the NETs showed progression with significant (according to RECIST criteria) growth of mesenterial and retroperitoneal lymphadenopathy, growth of hepatic metastases as well as newly emerged malignant pleural effusion. Urinary 5-HIAA levels also increased from 485 μmol/24 h to 1053 μmol/24 h (ref <50 μmol/24 h). Plasma chromogranine A was not measured in the past, but it was 317 ng/ml at the time of diagnosis of MG. The patient was started on octreotide therapy. Unfortunately, shortly after starting octreotide, the myasthenic symptoms worsened. Other provoking factors were excluded. The severity of symptoms required hospital admission. Octreotide was stopped and prednisolone was started together with further increase in the dose of pyridostigmine to six times daily, 50 mg. This led to reduction of symptoms. After tapering of the prednisolone, octreotide was reintroduced without further deterioration of the myasthenia. Currently, the NETs are only treated with octreotide that has caused a significant reduction in tumour markers to near-normal values as well as reduction of symptoms and radiologically stable disease. Of interest, in parallel with decreasing tumour markers, the myasthenic symptoms have disappeared and pyridostigmine could be stopped. To this date, no myasthenic symptoms have reappeared.

Patient B developed side effects of diarrhoea, for which the pyridostigmine dose had to be lowered to 60 mg two times daily with persistent relief of myasthenia. Currently, peptide receptor radiotherapy with lutetium-177 octreotate is being considered for the treatment of the NET metastases.

## Discussion

We present two patients with an ENETS stage IV NET of the small intestine with paraneoplastic MG occurring simultaneously with progression of the tumour. To our knowledge, this association was only described once in the current medical literature (3).

Of course, MG as a paraneoplastic phenomenon of thymoma is a widely recognised association. Next to thymic neoplasms, NETs of the lung (1) as well as pancreatic NETs (2) and haematological malignancies (4) have been described to coincide with MG.

### Neuroendocrine tumours

NETs are rare, with an estimated incidence in the Western world of ∼2/100 000 (5). The main primary sites are the lungs, gastrointestinal tract and pancreas (5) (6). These tumours are capable of secreting various bioactive substances that can cause symptoms depending on the amount and type of substance secreted, and on the presence of hepatic metastases. The most well-known clinical presentation is the carcinoid syndrome (7). As they are often asymptomatic for a long period of time, NETs have often already metastasised at the moment of diagnosis (5) (6). This implicates that the best treatment option, being complete surgical resection, is generally not feasible for most patients. In the presence of extensive metastases, there are several palliative pharmacotherapeutic options.

Most commonly used are somatostatin (sst) analogues that mainly give relief of symptoms. They also may have minor cytostatic effects, thereby improving progression-free survival (8).

Secondly, molecular targeted therapies with everolimus or sunitinib are very promising but these drugs are not yet approved for the treatment of gastrointestinal NETs (9).

### Myasthenia gravis

Although being a rare disease, MG is the most common disorder of the neuromuscular junction. The prevalence is estimated to be 10–15 per 100 000 (10). It is an autoimmune disease affecting the neuromuscular junction, in which antibodies against AChRs play an important role, but other antibodies can also be found (11). EMG is another tool to support the diagnosis of MG. Myasthenia can be exacerbated by various stressors among which are many drugs. The natural course of MG is very variable and unpredictable (10). The keystone of treatment is acetylcholinesterase inhibition by pyridostigmine. When MG occurs in the presence of a neoplasm, it is considered paraneoplastic. The current concept of pathogenesis of paraneoplastic MG is an immune response arising against epitopes expressed on tumour cells, with cross-reactivity to components of the neuromuscular junction, primarily AChRs (11). However, most research on this subject was performed in thymomas. In other tumours, the exact mechanism is not known. Clinically, paraneoplastic MG occurs in older persons when compared with classic idiopathic MG. It generally tends to be more severe and more frequently involves bulbar, respiratory and neck muscles. AChR antibodies are virtually always present in paraneoplastic MG but in lower titres when compared with non-paraneoplastic MG. Furthermore, 15% is seronegative at the time of diagnosis. Antibodies against striated muscle are also more prevalent (11). It is important to distinguish MG from Lambert–Eaton myasthenic syndrome, in which there are antibodies against presynaptic calcium channels (anti-VGCC) and which more frequently occurs as a paraneoplastic phenomenon to solid, non-thymic tumours and mainly with non-small cell lung carcinoma (1). The question still remains whether the introduction of octreotide was causally related to the deterioration of myasthenic symptoms in our first patient. Octreotide is a sst analogue, which is a neurotransmitter with a wide variety of actions in the CNS as well as in the autonomic nervous system. However, there is no current evidence that octreotide acts on the neuromuscular junction. Moreover, the second patient did not seem to have an interaction between octreotide and myasthenia. We can therefore only speculate, but it does not seem very likely that the worsening of myasthenic symptoms in the first patient was directly related to the octreotide. Still, in a potential new patient, we consider it to be important to start with a short-acting octreotide and to monitor muscle weakness/peak flow volumes. The second caveat in treating patients with paraneoplastic MG with gastrointestinal NETs is the fact that the most common adverse effect of pyridostigmine is diarrhoea, which often is present in patients with NETs. Finding the right ‘cocktail’ of octreotide and pyridostigmine can be challenging, as seen in the second patient.

## Patient consent

Written informed consent has been obtained from both patients.

## Author contribution statement

M A W Hermans drafted the main part of the article, acquisitioned data from both patients and performed a literature search. B M L Stelten drafted part of the article and was also responsible for data acquisition and part of the literature search. H R Haak contributed to the conception of the article and revised the manuscript critically. W W de Herder is the treating physician of patient B and revised the manuscript critically on multiple occasions. M W Dercksen is the treating physician of patient A, developed the conception of the article and also revised the manuscript critically on multiple occasions. All authors gave final approval of the version to be submitted.
